# Efficacy and safety of the Shugan Jieyu capsule as a complementary treatment for functional dyspepsia: A systematic review and meta-analysis

**DOI:** 10.1097/MD.0000000000044058

**Published:** 2025-08-29

**Authors:** Shuangli Peng, Yamei Ge, Huiyun Pu, Ping Yang, Hongci Chen

**Affiliations:** a Department of Traditional Chinese Medicine, Renmin Hospital of Wuhan University, Wuhan, China; b School of Chinese Medicine, Hubei University of Traditional Chinese Medicine, Wuhan, Hubei, China; c Department of Geriatrics, Hubei Provincial Hospital of Traditional Chinese Medicine, Wuhan, China.

**Keywords:** efficacy, functional dyspepsia, meta-analysis, safety, Shugan Jieyu capsule

## Abstract

**Background:**

To evaluate the efficacy and safety of the Shugan Jieyu capsule as a complementary treatment for functional dyspepsia (FD).

**Methods:**

Seven electronic databases were searched for randomized controlled trials (RCTs) on Shugan Jieyu capsule treatment for FD with a scientifically rigorous search strategy. The quality of the literature was assessed using the Risk of Bias 2 tool, a meta-analysis was performed with RevMan v5.3 and Stata 12.0.

**Results:**

Thirty-two RCTs containing 3582 participants were included in the meta-analysis. The combination of the Shugan Jieyu capsule with Western medications (WM) was more efficacious than treatment with WM alone (relative risk [RR] = 1.29, 95% confidence interval [CI]: [1.23, 1.35], *P* < .00001) and had comparable safety (RR = 1.0, 95% CI: [0.68, 1.47], *P* = .99). This combination also reduced the rate of disease recurrence (RR = 0.24, 95% CI: [0.14, 0.40], *P* < .00001) and patients’ gastrointestinal symptom scores (standardized mean difference [SMD] = −1.59, 95% CI: [−2.00, −1.18], *P* < .00001).

**Conclusion:**

While current evidence suggests SG as a complementary therapy may enhance FD treatment efficacy without increasing safety risks, these findings are constrained by the low methodological quality of included studies and exclusive derivation from Chinese populations. Definitive conclusions require future high-quality, multicenter RCTs with standardized outcome measures and diverse ethnic cohorts.

## 1. Introduction

Functional dyspepsia (FD) has been redefined as a disorder of the gut–brain interaction (DGBI), with 2 subtypes^[1]^: epigastric pain syndrome and postprandial distress syndrome. Symptoms include persistent and recurrent retrosternal pain, upper abdominal discomfort, postprandial fullness, early satiety, belching, nausea, acid reflux, heartburn, and constipation or diarrhea.^[2]^ The prevalence of FD varies across regions, with a global prevalence of 2.4% to 12.3% according to diagnoses using the latest Rome IV diagnostic criteria.^[3]^ Approximately 80% of patients with dyspepsia are diagnosed with FD.^[4]^ The high prevalence leads to significant healthcare costs,^[5]^ yet the therapeutic efficacy is not satisfactory.^[3]^ Furthermore, FD severely reduces people’s quality of life,^[6]^ and anxiety and depression are common in FD patients.^[7]^ As a result, people are increasingly emphasizing the gut–brain communication.^[8]^ Apart from common digestive system medications such as proton pump inhibitors (PPIs), histamine-2-receptor antagonists (H2RAs), gastrointestinal motility drugs, anti-Helicobacter pylori drugs,^[9]^ antidepressants are also being recognized for the treatment of FD,^[10]^ and in the latest British society of gastroenterology guidelines, tricyclic antidepressants are recommended as second-line treatment for patients with FD.^[3]^ However, it is regrettable that the guidelines did not take into account that some botanical drugs may treat FD through their potential antidepressant effects.

The Shugan Jieyu capsule (SG) is prepared from 2 botanical drugs, *Hypericum perforatum* L. [Hypericaceae; Hyperici herba] and *Eleutherococcus senticosus* (Rupr. & Maxim.) Maxim. [Araliaceae; Acanthopanacis radix et rhizoma seu caulis]. SG has been widely used in China for the treatment of depression.^[11]^ It contains various active components such as hypericin, pseudohypericin, quercetin, eleutheroside B, eleutheroside E, and others. These ingredients exert antidepressant effects by regulating the hypothalamic–pituitary–adrenal axis, affecting the monoaminergic system, and increasing brain-derived neurotrophic factor levels and their anti-inflammatory properties,^[12]^ compared to single-component WM, this indicates that botanical drugs can treat diseases through a multi-components, multi-target, and multi-pathway intervention mechanism.^[13]^ Information about SG, including its origin, components, description, extraction process, effects, and medical uses, is provided in Supplementary Material S1 (Supplemental Digital Content, https://links.lww.com/MD/P737), and the primary active constituents of SG are outlined in Supplementary Material S2 (Supplemental Digital Content, https://links.lww.com/MD/P737).

In fact, 3 prior meta-analyses^[14–16]^ reported that SG can effectively treat FD; however, these 3 meta-analyses had substantial limitations. Firstly, in Guo study, the choice of odds ratio (OR) as the effect size for binary outcome variables might be considered unwise. When the occurrence rate of the outcome exceeds 10%, the OR tends to overestimate the intervention’s effect compared to the relative risk (RR), resulting in inflated effect estimates.^[17]^ Tang research included flawed original studies, evident reporting errors, and publication bias. Cheng study revealed inconsistencies between the inclusion criteria and the selected studies. More significantly, their approach of addressing heterogeneity by excluding specific studies is questionable.^[18]^ Additionally, relying solely on funnel plots to assess publication bias is highly subjective, and the conclusions drawn without conducting sensitivity analysis are not robust. Secondly, these meta-analyses were not conducted according to the preferred reporting items for systematic review and meta-analysis (PRISMA) statement,^[19]^ and the meta-analyses were not registered. Furthermore, including only studies published in Chinese^[15,16]^ is not conducive to the dissemination of study results. Finally, these meta-analyses included only randomized controlled trials (RCTs) published before May 15, 2020, so an updated meta-analysis including recent studies is needed. Therefore, we conducted our meta-analysis with a rigorous design and with the aim of achieving stable and reliable results.

## 2. Methods

This study was based on the PRISMA statement and was registered with the International Prospective Register of Systematic Reviews (PROSPERO) (registration number: CRD42023414203) to ensure methodological rigor. As this was a meta-analysis, the need for ethics approval and informed consent were waived.

### 2.1. Literature search

Two researchers (Shuangli Peng and Yamei Ge) independently conducted the literature search. The sources searched included the PubMed, Cochrane Library, Embase, China National Knowledge Infrastructure, China biology medicine, Wanfang data knowledge service platform, and Chongqing VIP databases. The search period was from October 20, 2008, to January 1, 2024, and studies that were published in Chinese or English were included. The search strategy was developed using a combination of keywords (MeSH terms) and free-text terms; the search terms included “*Shugan Jieyu*,” “functional dyspepsia” and their synonyms. Boolean operators (AND, OR, and NOT) were used to combine search terms. The search strategy was adjusted for each database to reflect its specific features, and the search field and all search strategies are provided in Supplementary Material S3 (Supplemental Digital Content, https://links.lww.com/MD/P737).

### 2.2. Study selection and data extraction

The identified records were screened according to predetermined inclusion and exclusion criteria by 2 researchers working independently, and a third researcher was consulted in the event of conflicting opinions. We developed the following inclusion and exclusion criteria:

The inclusion criteria for studies were as follows: Types of study: We incorporated solely parallel-group RCTs. Types of participants: Patients should have a definitive diagnosis, such as the Rome diagnostic criteria or the Chinese expert consensus. There are no specific requirements regarding the patient’s age, gender, race, nationality, disease subtype, or the presence of comorbid anxiety or depression. Types of interventions: The control group was administered either a placebo or conventional Western medications (WM) as mentioned, such as PPIs, H2RAs, gastrointestinal motility drugs, anti-*H pylori* drugs, and antidepressants. It is essential to provide a detailed description of the drug dosage, frequency, and duration of treatment. The intervention strategy for the experimental group involved combining SG with the aforementioned conventional WM. The treatment duration for both groups was mandated to be a minimum of 4 weeks. Types of outcome measures: The primary outcomes are clinical efficacy and safety. Clinical efficacy encompasses the proportions of patients who experienced complete recovery, significant improvement, and moderate efficacy. This efficacy can be qualitatively elucidated by detailing the degree of symptom amelioration or quantitatively discerned based on the percentage reduction in scores from relevant assessment scales pre- and posttreatment. Safety refers to the comparison of the frequency of adverse reactions between the control group and the experimental group. Secondary outcomes include the disease recurrence rate and gastrointestinal symptom scores.

The exclusion criteria for studies were as follows: The studies did not have an RCT design; the experimental group was not permitted to use other Chinese patent medicines or decoctions, Chinese medicine injections, massage, Tai Chi, Qigong, acupuncture, or moxibustion; and the presence of complete outcome data, but these data appeared incorrect.

Two researchers (HP and PY) independently extracted all data from the studies, including first author, publication year, study region, diagnostic criteria, sample size, mean age, sex ratios, medication regimens, and outcomes. If the original data were missing or unable to be extracted, we requested these data from the corresponding author by email. If we were unable to contact the corresponding author, we discussed these studies in the Results section.

### 2.3. Quality assessment

The Risk of Bias 2 tool (RoB2), which is used to assess RoB in RCTs,^[20]^ was used to assess the quality of RCTs included in this meta-analysis; studies were labeled “low risk of bias”, “some concerns” or “high risk of bias”. This assessment was performed independently by 2 researchers (HC and SP) to ensure the consistency of the meta-analysis results.

### 2.4. Data analysis

We utilized RevMan 5.3 to analyze data from all included RCTs. Dichotomous variables are presented as the RR, and continuous variables with the same units are expressed as the mean difference (MD); otherwise, they are expressed as the standardized mean difference (SMD), with a 95% confidence interval (CI).

Heterogeneity among studies was quantified using the *I*^2^ statistic following the guidelines provided in the Cochrane Handbook. Heterogeneity was classified into 4 levels: low (0 < *I*^2^ < 40%), moderate (30% <*I*^2^ < 60%), substantial (50% < *I*^2^ < 90%), or high (75% < *I*^2^ < 100%). Importantly, due to clinical and methodological diversity, statistical heterogeneity is often present in meta-analyses.^[21]^ To better account for potential heterogeneity, we prefer the use of the random-effects model for assessing intervention effects rather than selecting between the fixed-effects model or random-effects model solely based on the degree of heterogeneity.

We conducted subgroup analyses based on treatment duration, mental illness, sample size, age, interventions, and drug dosages to examine the significance of differences among various subgroups and explore potential heterogeneity. Furthermore, we employed Stata 12.0 software to assess the stability of our primary outcomes using the leave-one-out method and the trim-and-fill method. Additionally, we utilized funnel plots, Begg test, and Egger test to identify publication bias.

## 3. Results

### 3.1. Characteristics of the included RCTs

**Figure 1.**
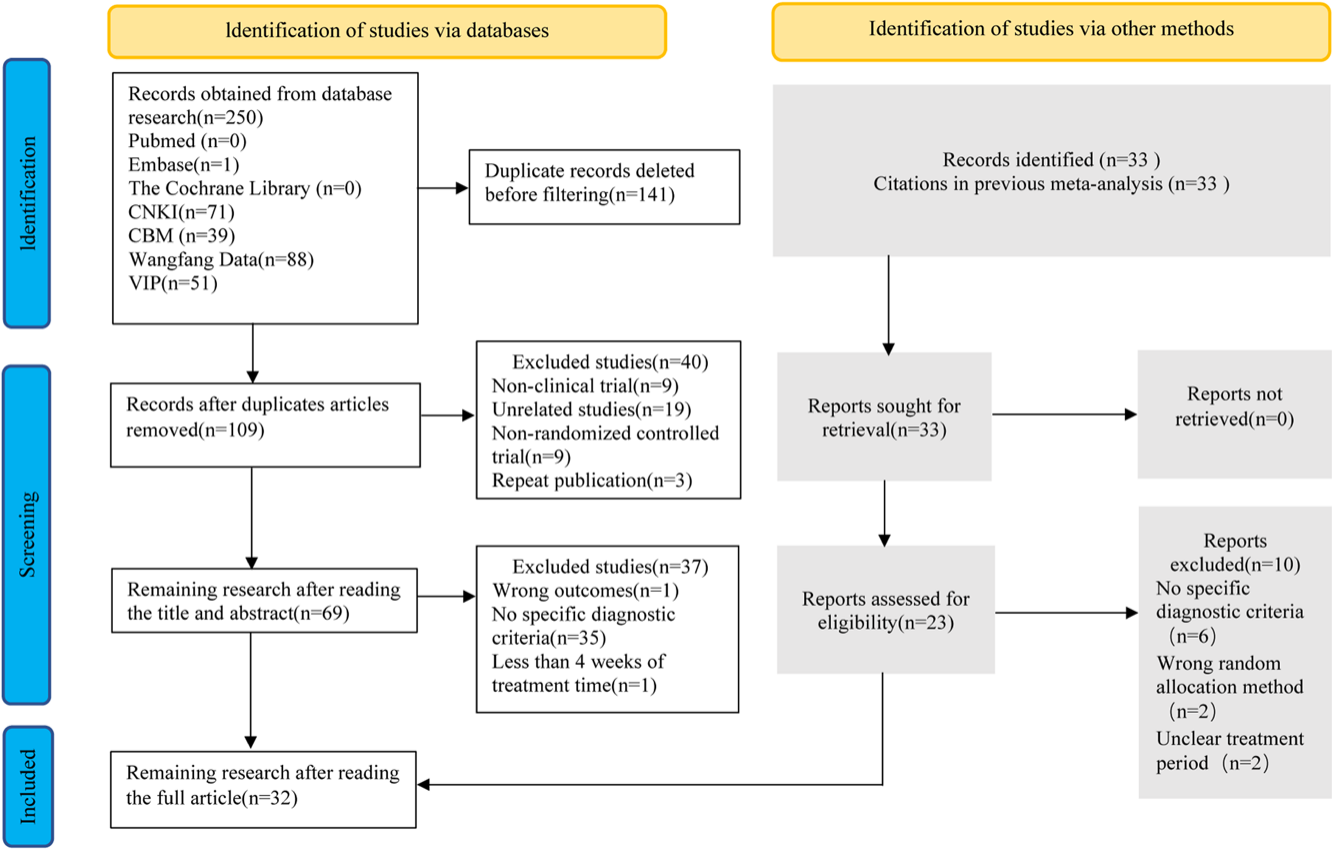
Flow chart.

Using the predefined search criteria, we initially retrieved 250 records; 109 remained after excluding duplicates, 69 remained after review of the titles, and 32 remained after review of the full text. We also included 23 studies after screening the studies included in previous meta-analyses, and these 23 studies were included in the 32 studies finally selected for inclusion in the present meta-analysis^[22–53]^ (Fig. 1). All 3582 participants were from China. Four studies^[25,27,40,50]^ did not specify sex ratios; the remaining 28 studies included 1423 males and 1737 females. The sample sizes of the treatment groups ranged from 30 to 255 participants, while those of the control groups ranged from 30 to 130 participants. The treatment duration ranged from 4 to 8 weeks. Commonly administered medications in the control groups included domperidone, mosapride, itopride, rabeprazole, pantoprazole, and compound digestive enzyme capsules. The treatment groups received concomitant administration of SG in addition to the treatment regimen used in the control groups. All studies had a 2-arm clinical trial design and utilized random allocation methods. Thirteen studies^[22,24,28,29,31,37,38,41,43,44,47,49,51]^ described the specific randomization approach used, such as the employment of random number tables. Furthermore, 2 studies^[23,34]^ implemented blinding procedures. However, allocation concealment methods were notably not reported in any study. The findings of all 32 studies are presented comprehensively. Detailed characteristics of the studies are outlined in Table 1.

**Table 1:** Characteristics of the included literature.

Study	Diagnostic criteria	Sample size	Age (yr)		Gender (M/F)		Treatment duration		Treatments (wk)	Medication regimen		Outcomes
		(T/C)	T	C	T	C	T	C		T	C	
He 2023^[24]^	Expert consensus	103/103	42.97 ± 8.74	43.13 ± 8.76	55/48	56/47	3.03 ± 0.90 (yr)	2.98 ± 0.87 (yr)	8	C + SG 0.72 g bid	Omeprazole (10 mg qd); Domperidone (10 mg tid)	*,†
Xu 2020^[47]^	Rome III	49/49	45.83 ± 7.16	46.21 ± 8.07	27/22	26/23	2.18 ± 0.34 (yr)	2.24 ± 0.68 (yr)	6	C + SG 0.72 g bid	Rabeprazole (20 mg qd); Domperidone (10 mg tid)	*,†,‡
Gan 2019^[23]^	Rome III	225/130	45.58 ± 9.52	43.56 ± 8.32	75/150	38/92	21.98 ± 12.58 (mo)	21.79 ± 11.39 (mo)	4	C + SG 0.72 g bid	Rabeprazole (20 mg qd); Domperidone (10 mg tid); Placebo (0.72 g bid)	*,†,‡
Wang 2019^[42]^	Rome III	30/30	54.50 ± 4.22	54.46 ± 4.16	13/17	14/16	8.9 ± 2.38 (yr)	8.56 ± 2.33 (yr)	8	C + SG 0.72 g bid	Mosapride (5 mg tid)	*,†,‡
Wang 2019^[41]^	Expert consensus	48/47	54.03 ± 5.73	53.96 ± 5.64	29/19	26/21	3.06 ± 0.39 (yr)	3.03 ± 0.41 (yr)	4	C + SG 0.72 g bid	Compound digestive enzymes (1–2# tid)	*,†
Hu 2019^[27]^	Rome III	50/50	NA	NA	NA	NA	NA	NA	4	C + SG 0.72 g bid	Rabeprazole (10 mg qd)	*,†
Li 2018^[37]^	Rome III	49/49	42.9 ± 6.1	43.1 ± 6.8	18/31	21/28	26.8 ± 10.7 (mo)	27.9 ± 11.4 (mo)	6	C + SG 0.72 g bid	Rabeprazole (20 mg qd); Domperidone (10 mg tid)	*
Shan 2018^[22]^	Rome III	35/33	71.2 ± 3.2	72.8 ± 2.9	17/18	14/19	NA	NA	6	C + SG 0.72 g bid	Magnesium aluminum carbonate (1.0 g tid)	*,†
Wang 2017^[43]^	Rome III	77/76	52.63 ± 3.86	53.76 ± 3.92	43/34	41/35	3.22 ± 1.07 (yr)	3.42 ± 1.14 (yr)	4	C + SG 0.72 g bid	Mosapride (5 mg tid); Paroxetine (20 mg qd)	*
Li 2017^[38]^	Rome III	55/55	45.8 ± 10.2	44.6 ± 9.8	24/31	26/29	3.5 ± 0.8 (yr)	3.2 ± 0.6 (yr)	4	C + SG 0.72 g bid	Mosapride (5 mg tid)	*,‡,§
Zhang 2017^[51]^	Rome II	49/49	39.47 ± 13.22	37.54 ± 12.03	35/14	31/18	10.66 ± 4.18 (mo)	9.58 ± 5.23 (mo)	4	C + SG 0.72 g bid	Trimebutine maleate tablets (100 mg tid)	*
Yang 2017^[49]^	Rome III	46/46	43 ± 7	43 ± 7	19/27	20/26	3.7 ± 1.3 (yr)	3.6 ± 1.3 (yr)	6	C + SG 0.72 g bid	Rabeprazole (10 mg bid); Mosapride (5 mg tid)	*,†
Hao 2011^[25]^	Rome III	60/60	NA	NA	NA	NA	NA	NA	4	C + SG 0.72 g bid	Trimebutine maleate tablets (100 mg tid); Compound azintamide enteric-coated tablets (100 mg tid)	*,†
Wu 2011^[26]^	Rome III	60/60	46	45	22/38	25/35	NA	NA	4	C + SG 0.72 g bid	Mosapride (5 mg tid)	*,†,§
Geng 2012^[29]^	Expert consensus	46/46	51.66 ± 4.65	51.74 ± 6.54	24/22	25/21	6.11 ± 4.53 (yr)	5.98 ± 4.57 (yr)	4	C + SG 0.72 g bid	Mosapride (5 mg tid)	*,†
Huang 2012^[32]^	Rome III	40/40	39.25 ± 12.33	38.50 ± 11.45	17/23	14/26	NA	NA	6	C + SG 0.72 g bid	Gastrointestinal prokinetics, acid suppressants, or gastric mucosal protective agents	*
Tan 2012^[40]^	Rome III	65/65	NA	NA	NA	NA	NA	NA	4	C + SG 0.72 g bid	Mosapride (5 mg tid)	*
Xi 2012^[45]^	Rome III	46/40	41.34 ± 1.72	39.86 ± 1.64	19/27	16/24	NA	NA	6	C + SG 0.72 g bid	Mosapride (5 mg tid)	*,†,‡
Zhang 2013^[52]^	Rome II	46/46	37.2	38.3	24/22	26/20	NA	NA	4	C + SG 0.72 g bid	Itopride (50 mg tid)	*
Xiao 2013^[46]^	Rome III	46/46	43.26	43.12	30/16	32/14	NA	NA	4	C + SG 1.28 g tid	Lansoprazole (40 mg qd); Mosapride (10 mg tid)	*
Li 2014^[36]^	Rome III	48/52	52.2 ± 11.3	53.1 ± 12.5	23/25	23/29	1.5 ± 0.3 (yr)	1.7 ± 0.4 (yr)	6	C + SG 0.72 g bid	Mosapride (5 mg tid)	*,†,§
Ji 2015^[33]^	Rome III	50/49	34.27 ± 3.0	37.12 ± 2.7	19/31	15/34	NA	NA	8	C + SG 0.72 g bid	Mosapride (10 mg tid)	*
Yang 2016^[48]^	Rome III	53/53	52.4 ± 2.7	52.1 ± 2.8	35/18	34/19	3.4 ± 1.2 (yr)	3.2 ± 1.1 (yr)	4	C + SG 0.72 g bid	Lansoprazole (30 mg qd); Mosapride (5 mg tid)	*,†
Li 2016^[34]^	Rome III	135/110	42.56 ± 8.23	42.61 ± 8.87	32/103	28/82	22.43 ± 11.96 (mo)	22.57 ± 12.10 (mo)	4	C + SG 0.72 g bid	Rabeprazole (20 mg qd); Domperidone (10 mg tid); Placebo (0.72 g bid)	*,†,‡
Li 2016^[35]^	Expert consensus	42/42	41.8 ± 9.1	42.3 ± 8.9	21/21	20/22	21.8 ± 5.4 (yr)	22.4 ± 5.6 (yr)	4	C + SG 0.36 g bid	Domperidone (10 mg tid)	*,†
Guo 2016^[31]^	Rome III	80/80	44.1 ± 6.7	43.6 ± 5.2	45/35	43/37	2.8 ± 0.9 (yr)	2.6 ± 0.8 (yr)	6	C + SG 1.28 g tid	Conventional treatment; Venlafaxine (75 mg qd)	†,§
Wang 2012^[44]^	Rome III	30/30	43.9	45	6/24	7/23	NA	NA	4	C + SG 0.72 g bid	Domperidone (10 mg tid)	*
Dong 2012^[28]^	Expert consensus	42/42	51.07 ± 6.08	51.49 ± 6.35	19/23	15/27	5.46 ± 4.59 (yr)	5.17 ± 4.39 (yr)	4	C + SG 0.72 g bid	Mosapride (5 mg tid)	*,§
Guo 2017^[30]^	Expert consensus	30/30	45.78 ± 12.15	46.33 ± 12.25	15/15	15/15	3.98 ± 2.15 (yr)	4.25 ± 2.07 (yr)	6	C + SG 0.72 g bid	Mosapride (5 mg tid)	*
Zou 2020^[53]^	Rome III	36/36	40.39 ± 3.02	40.67 ± 5.16	21/15	20/16	1.10 ± 0.12 (yr)	1.08 ± 0.13 (yr)	4	C + SG 0.72 g bid	Compound digestive enzymes (2# tid)	†
Liu 2018^[39]^	Rome III	48/47	NA	NA	10/38	15/32	NA	NA	6	C + SG 0.72 g bid	Rabeprazole (10 mg qd) or Mosapride (5 mg tid)	†
Zhang 2018^[50]^	Rome III	36/36	NA	NA	NA	NA	NA	NA	4	C + SG 0.72 g bid	Mosapride (5 mg tid)	*

bid = twice a day, C = control group, F = female, M = male, NA = not applicable, qd = once a day, T = treatment group, tid = 3 times a day.

* Clinical efficacy.

† Safety.

‡ Gastrointestinal symptom scores.

§ Recurrence rate.

### 3.2. Quality of the included RCTs

**Figure 2.**
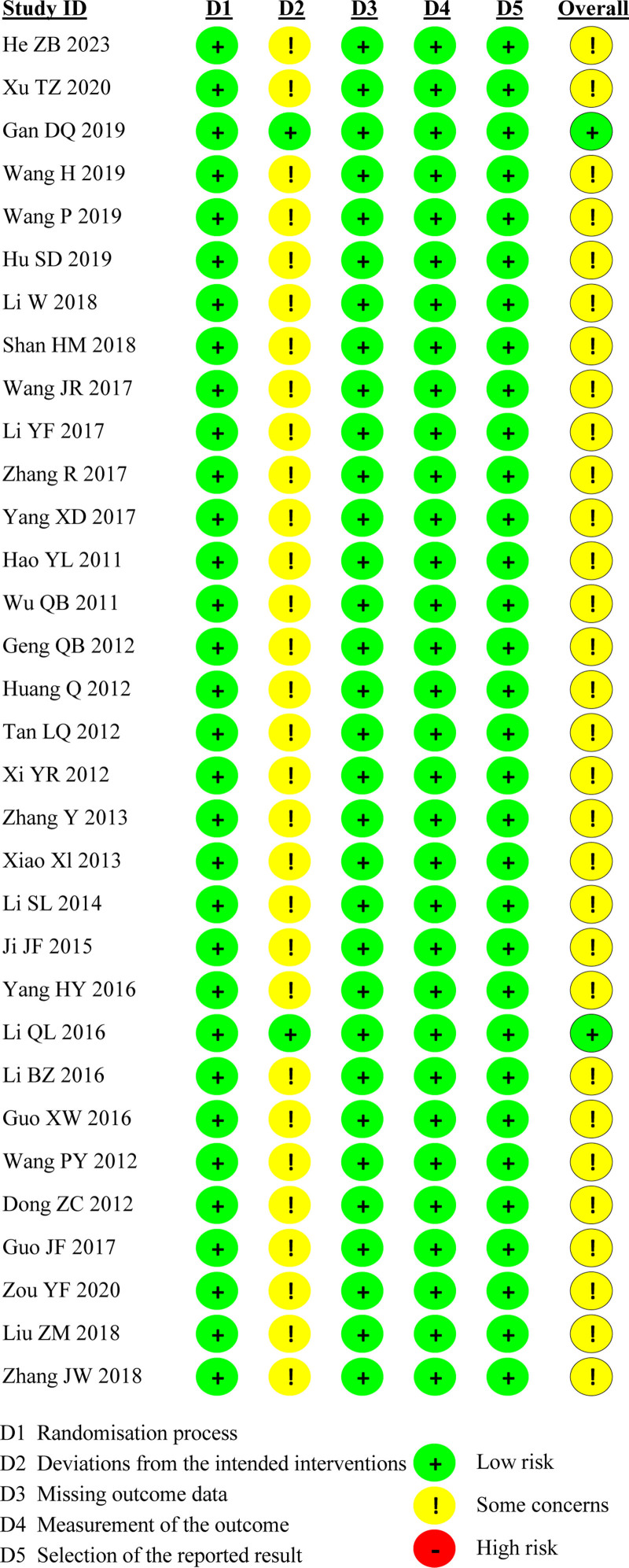
Risk of bias.

**Figure 3.**
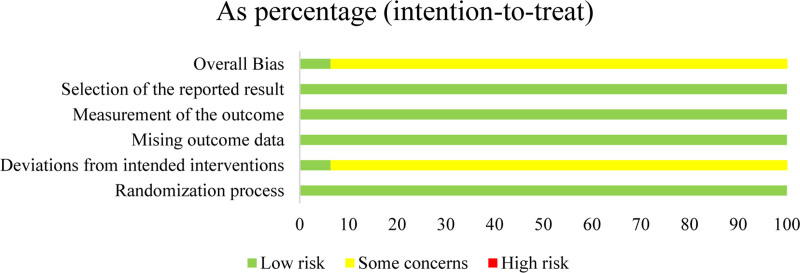
Risk of bias summary.

The RoB2 tool, which assesses the quality of 32 RCTs, was employed to evaluate various aspects, including the randomization process, deviations from intended interventions, missing outcome data, measurement of the outcome, and selection of the reported results. The findings indicated a low RoB in the randomization process, missing outcome data, selection of the reported result, and measurement of the outcome, and some concerns regarding deviations from intended interventions. In the overall assessment, 2 RCTs^[23,34]^ were classified as low risk, 30 as having some concerns, and none as high risk (Figs. 2 and 3).

### 3.3. Marked clinical efficacy

**Figure 4.**
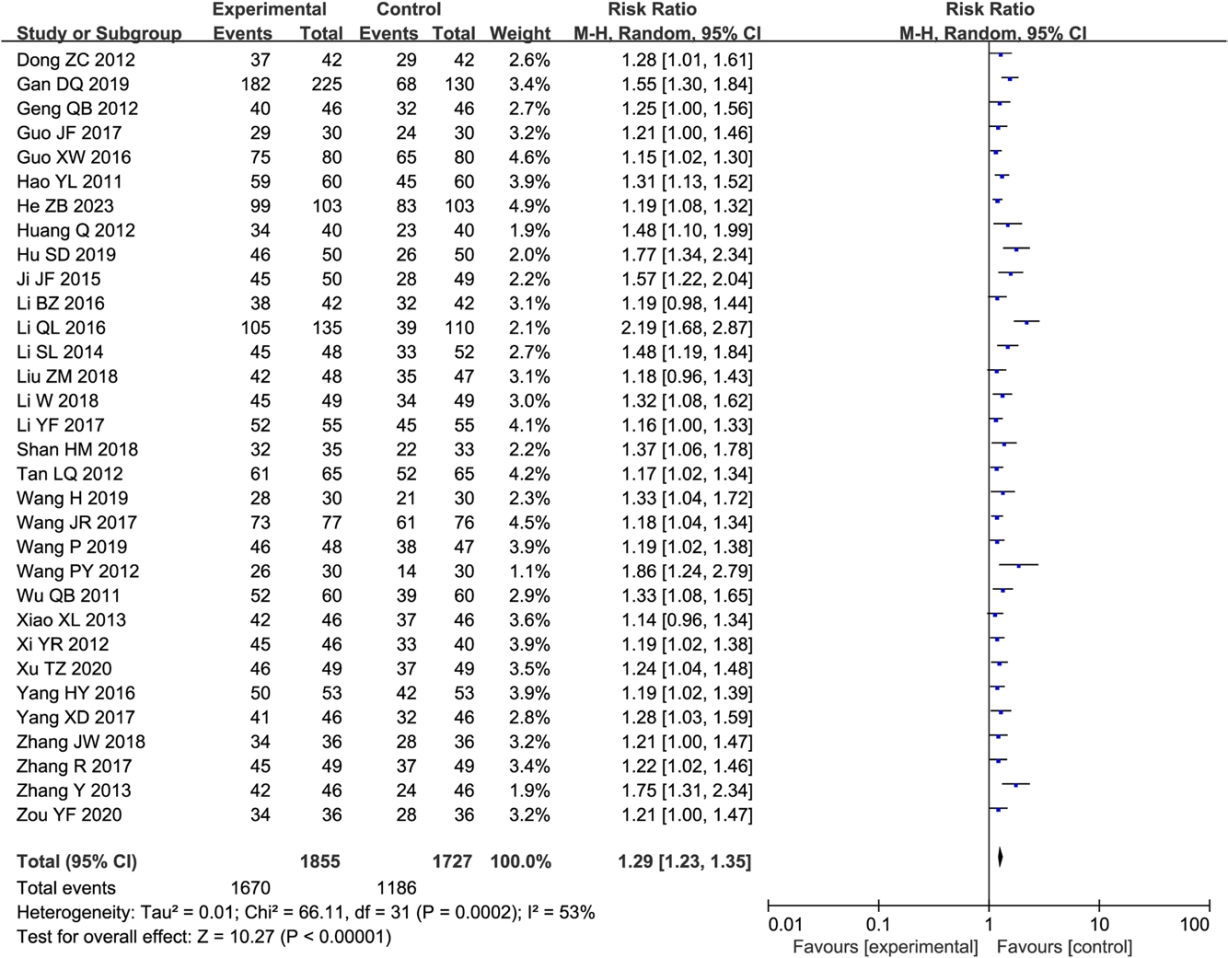
Forest plot of clinical efficacy.

Thirty-two studies including 3582 participants explored clinical efficacy, and the RR was 1.29 with a 95% CI of [1.23, 1.35], indicating a significant association (*P* < .00001). There was moderate heterogeneity among studies (*I*^2^ = 53%) (Fig. 4). Subsequently, detailed subgroup analyses were conducted across various dimensions, including treatment duration, mental illness, sample size, age, drug dosages and interventions. Although no sources of heterogeneity were identified, each subgroup result was statistically significant. The outcomes of these subgroup analyses are summarized in Table 2.

**Table 2:** Subgroup analysis of clinical efficacy.

Characteristics	Subgroups	RCTs	Participants	*I*^2^ (%)	*P* (het)	RR	95% CI	*P*
Treatment duration	4 wk	19	2253	37	.05	1.28	1.21–1.34	<.00001
	6 wk	10	1090	77	<.0001	1.35	1.18–1.54	<.00001
	8 wk	3	239	0	.96	1.20	1.07–1.34	.0001
Mental illness	No depression	9	938	0	.7	1.24	1.17–1.32	<.00001
	Depression	23	2644	65	<.0001	1.31	1.23–1.40	<.00001
Sample size	60–89	10	726	0	.583	1.25	1.17–1.34	<.00001
	90–119	14	1367	42.6	.046	1.28	1.20–1.37	<.00001
	120–149	3	370	0	.443	1.25	1.14–1.37	<.00001
	150–179	2	313	0	.789	1.17	1.07–1.27	<.00001
	≥180	3	806	93.4	<.00001	1.57	1.07–2.32	.022
Age (yr)	<45	14	1564	71.4	<.00001	1.33	1.21–1.46	<.00001
	≥45	13	1501	15.6	.287	1.26	1.19–1.33	<.00001
Drug dosages	0.36 g bid	2	252	0	.876	1.15	1.04–1.26	.006
	0.72 g bid	29	3246	54.6	<.00001	1.31	1.24–1.38	<.00001
Interventions	SG + GMD vs GMD	14	1249	35	.1	1.28	1.20–1.37	<.00001
	SG + PPI + GMD vs PPI + GMD	8	1292	79	<.0001	1.33	1.17–1.53	<.0001
	SG + CDE vs CDE	2	167	0	.85	1.20	1.06–1.35	=.003

bid = twice a day, CDE = compound digestive enzymes, CI = confidence interval, GMD = gastrointestinal motility drug, PPIs = proton pump inhibitors, RCTs = randomized controlled trials, RR = relative risk, SG = Shugan Jieyu capsule.

### 3.4. Equivalent safety

**Figure 5.**
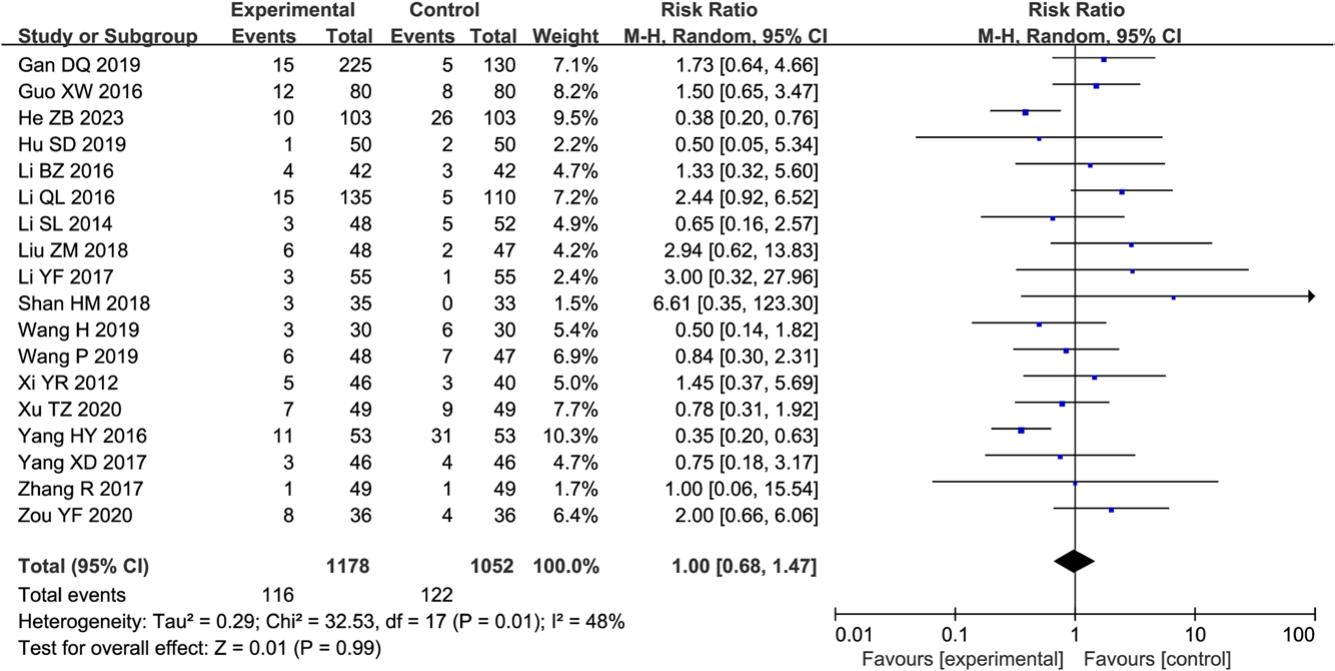
Forest plot of safety.

Safety was assessed in 18 studies^[22–24,27,31,34–36,38,39,41,42,45,47–49,51,53]^ with a total sample size of 2230 participants. Common adverse events include dry mouth, nausea, constipation, dizziness, increased defecation frequency, sleep disorders, diarrhea, gastrointestinal discomfort, headache, and loss of appetite. All subjects exhibited good tolerance to adverse events, and none withdrew due to adverse events. Detailed information regarding adverse events can be found in Table 3. The results based on the random-effects model revealed an estimated RR of 1.00, with a 95% CI of 0.68 to 1.47, but the difference was not statistically significant (*P* = .99 > 0.05) (Fig. 5). There was moderate heterogeneity among studies (*I*^2^ = 48%). Subsequently, we performed subgroup analyses considering treatment duration, mental illness, sample size, age and interventions. Detailed results of these subgroup analyses are presented in Table 4.

**Table 3:** Adverse events of included RCTs.

	Experimental group		Control group	
	No. of AEs	No. of RCTs	No. of AEs	No. of RCTs
Dry mouth	10	7	12	6
Nausea	12	6	15	5
Constipation	11	6	17	6
Dizziness	14	6	23	6
Increased defecation frequency	14	5	10	5
Sleep disorders	7	3	15	2
Diarrhea	1	1	4	3
Gastrointestinal discomfort	5	3	0	0
Headache	7	2	3	2
Loss of appetite	4	2	0	0

AEs = adverse events, RCTs = randomized controlled trials.

**Table 4:** Subgroup analysis of safety.

Characteristics	Subgroups	RCTs	Participants	*I*^2^ (%)	*P* (het)	RR	95% CI	*P*
Treatments	4 wk	9	1052	71	.0005	0.49	0.26–0.95	.03
	6 wk	7	699	0	.54	1.18	0.74–1.87	.49
	8 wk	2	266	0	.72	0.41	0.22–0.74	.003
Mental illness	No depression	15	1938	55	.006	0.96	0.63–1.46	.86
	Depression	3	292	0	.79	1.55	0.51–4.68	.44
Sample size	60–89	5	370	0	.42	1.32	0.71–2.47	.39
	90–119	9	478	22.9	.24	0.71	0.44–1.14	.16
	>120	4	966	76.8	.005	1.20	0.49–2.92	.70
Age	<45	8	1043	49.4%	.054	1.161	0.657–2.052	.608
	≥45	8	992	46.7%	.069	0.797	0.458–1.388	.423
Interventions	SG + GMD vs GMD	5	440	0	.58	0.96	0.50–1.85	.91
	SG + PPI + GMD vs PPI + GMD	7	1197	72	.002	0.90	0.46–1.74	.94

CI = confidence interval, GMD = gastrointestinal motility drug, PPIs = proton pump inhibitors, RCTs = randomized controlled trials, RR = relative risk, SG = Shugan Jieyu capsule.

### 3.5. Low recurrence rate

**Figure 6.**
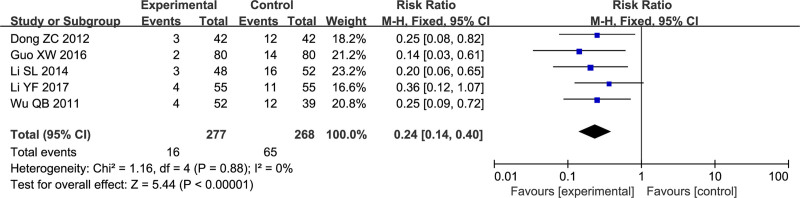
Forest plot of recurrence rates.

Five studies^[26,28,31,36,38]^ with 545 participants examined recurrence rates. In Dong^[28]^ study, the experimental group had a 7.14% recurrence rate, while the control group had a 28.57% recurrence rate. Guo^[31]^ study reported 2.5% in the experimental group and 17.5% in the control group. Li^[36]^ research showed 6.25% for the experimental group and 30.76% for the control. Li^[38]^ study found 7.27% and 20%, respectively, and Wu^[26]^ study indicated 7.69% for the experimental group and 30.76% for the control. The results were RR = 0.24, 95% CI: [0.14, 0.40]. The difference was statistically significant (*P* < .00001) (Fig. 6). There was no apparent heterogeneity among the studies (*I*^2^ = 0%).

### 3.6. Reduced gastrointestinal symptom scores

**Figure 7.**
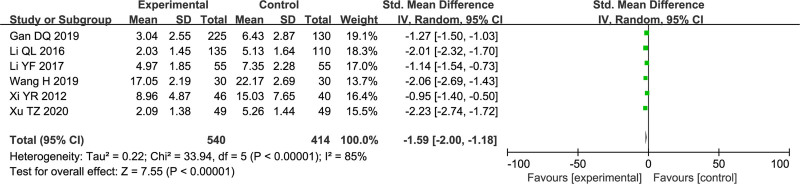
Forest plot of gastrointestinal symptom scores.

Six studies^[23,34,38,42,45,47]^ explored gastrointestinal symptom scores and included 954 participants. Based on the analysis, SMD = −1.59, 95% CI: [−2.00, −1.18], the difference was statistically significant (*P* < .00001) (Fig. 7), and there was high heterogeneity among the studies (*I*^2^ = 85%).

### 3.7. Sensitivity analysis

**Figure 8.**
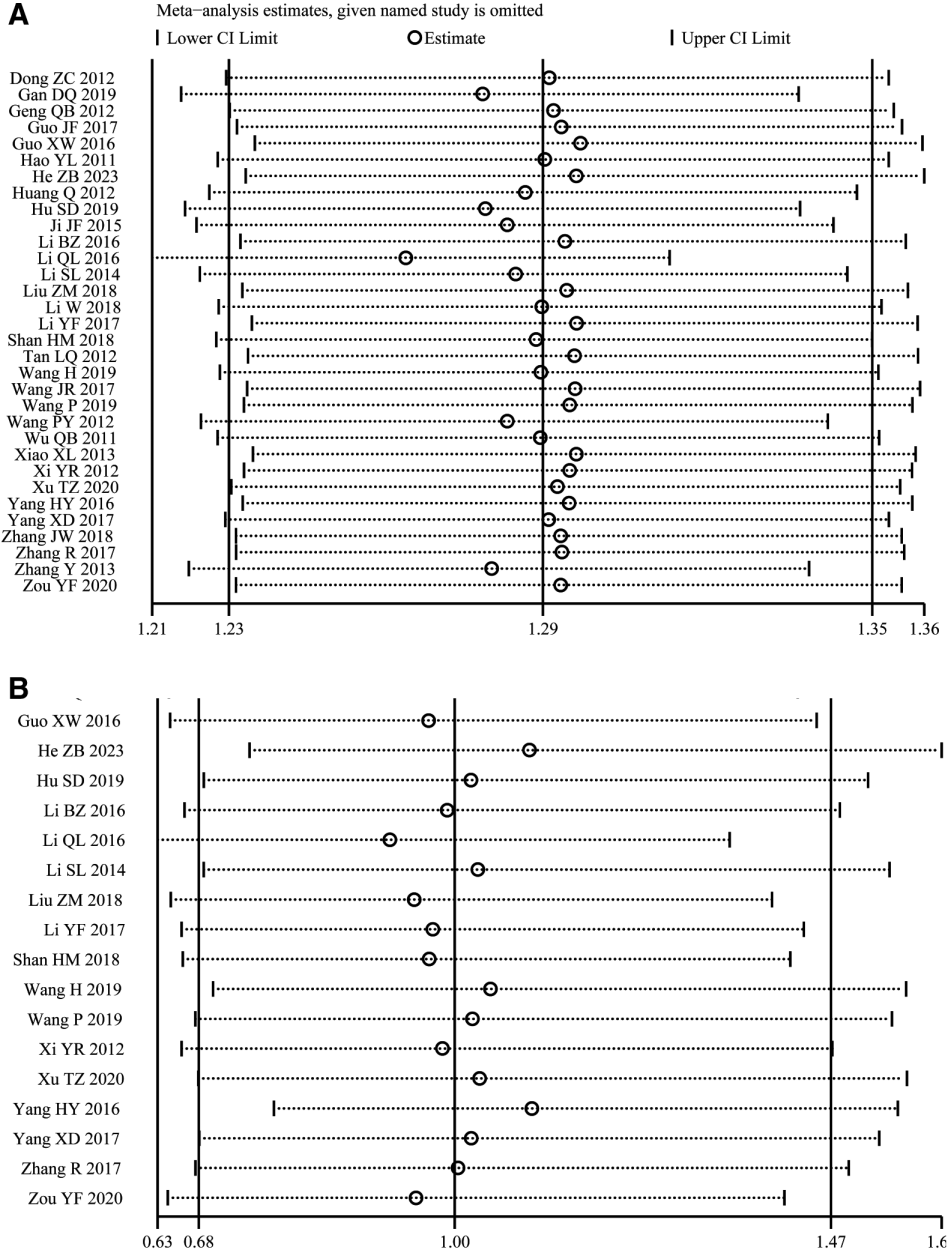
Sensitivity analysis. (A) Sensitivity analysis of clinical efficacy using the leave-one-out method. (B) Sensitivity analysis of safety outcomes using the leave-one-out method.

We performed sensitivity analyses of clinical efficacy and safety. With the leave-one-out method, we observed that the effect sizes remained within the credible interval regardless of which study was excluded (Fig. 8A and B). This observation indicates the robustness of the results.

### 3.8. Publication bias

**Figure 9.**
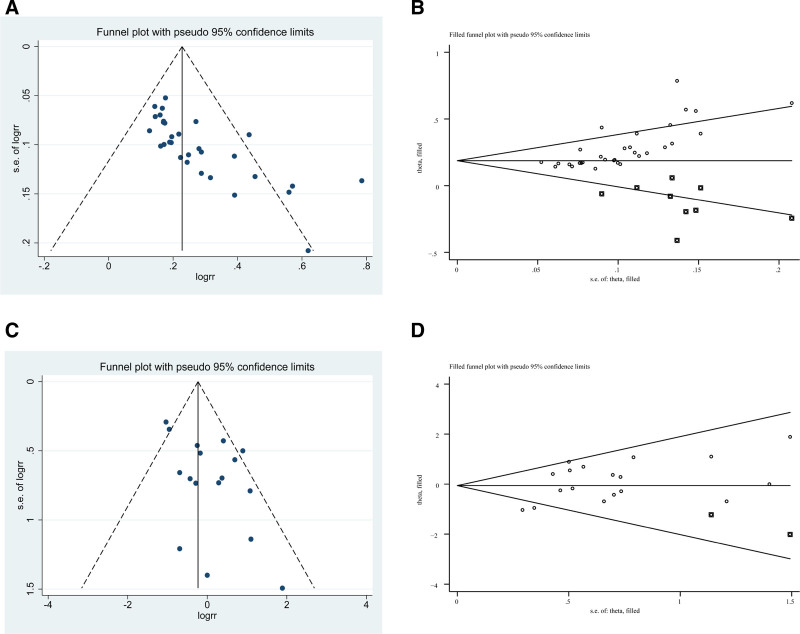
Funnel plots assessing publication bias and the effect of trim-and-fill adjustment. (A) Clinical efficacy (pre-trim-and-fill). (B) Clinical efficacy after trim-and-fill adjustment. (C) Safety outcomes (pre-trim-and-fill). (D) Safety outcomes after trim-and-fill adjustment.

The asymmetric funnel plots suggested publication bias regarding clinical efficacy (Fig. 9A), and Begg test and Egger test suggested the presence of publication bias (*P* < .05). After applying the trim-and-fill method, 9 missing RCTs were identified on the left side of the funnel plot; however, the effect size values did not change significantly, suggesting that the results were robust (Fig. 9B). Similarly, the funnel plot on safety was asymmetric (Fig. 9C). Begg test indicated the absence of publication bias (*P* > .05), while Egger test suggested the presence of publication bias (*P* < .05). The application of the trim-and-fill method revealed 2 missing RCTs on the left side of the funnel plot, but the effect size values did not change significantly, suggesting that the results were robust (Fig. 9D).

## 4. Discussion

### 4.1. Interpretation of the results

Clinical efficacy was one of the primary outcomes of the present meta-analysis, and pooled analysis of 32 studies revealed that the combination of SG and WM was more effective for treating FD than WM alone (RR = 1.29, 95% CI: [1.23, 1.35]), which is consistent with previous studies.^[15,16]^ The *I*^2^ value was 53%, indicating moderate heterogeneity among the studies.^[54]^ After ruling out errors in the raw data, we conducted a comprehensive subgroup analysis based on treatment duration, mental illness, sample size, age, interventions and drug dosages. Subgroup analysis indicated that as a complementary therapy, SG can enhance the therapeutic effect by 1.20 times when used in conjunction with compound digestive enzymes. Furthermore, when combined with gastrointestinal motility drugs, the efficacy increases to 1.28 times the original effect. The most pronounced enhancement, however, is observed when SG is administered with both PPIs and gastrointestinal motility drugs, resulting in an efficacy increase to 1.33 times the original effect. Additionally, the dose of 0.72 g twice daily demonstrates more pronounced efficacy compared to the dose of 0.36 grams twice daily. Treatment durations of 4, 6, and 8 weeks all provide benefits to patients, with 6 weeks appearing to be the optimal duration, while efficacy begins to decline at 8 weeks. Furthermore, patients younger than 45 seem to benefit more. The British society of gastroenterology guidelines for the management of FD recommend antidepressant medication as a second-line treatment. Experts have suggested initiating clinical trials for the early administration of antidepressants in FD patients without concurrent depression. In our subgroup analysis, we categorized patients into 2 groups: those with and those without comorbid depression. Notably, compared to the depressed group, the nondepressed group had an RR of 1.24 (95% CI: [1.17–1.32], *P* < .00001], supporting the idea that early commencement of SG treatment can be beneficial for FD patients. Therefore, we speculate that early initiation of antidepressant treatment may benefit patients with FD, but this needs to be confirmed by studies with larger sample sizes. The stability of the results was verified by sensitivity analysis, which used the leave-one-out method.^[55]^ Asymmetric funnel plots and results from Begg tests and Egger tests suggested publication bias, but subsequent corrected funnel plots based on the trim-and-fill method suggested nonsignificant changes in effect sizes, further confirming the robustness of the results.^[56]^

Safety was the other primary outcome of this analysis, and a meta-analysis of 18 studies showed that SG combined with WM had effects comparable to those of WM treatment alone (RR = 1.00, 95% CI: [0.68, 1.47], *P* = .99). We performed subgroup analyses of treatment duration, mental illness, sample size, age, interventions and drug dosages. Interestingly, the treatment was safer than the control treatment for a treatment duration of 4 or 8 weeks (*P* < .05), while the remaining subgroup analyses suggested that the safety was comparable between the 2 groups. Although sensitivity analyses suggested that the results were robust, the funnel plots were asymmetric; Begg test did not indicate publication bias, whereas Egger test (which is more sensitive) suggested the presence of publication bias (*P* < .05).^[57]^ The effect sizes changed only slightly after applying the trim-and-fill method, indicating that the results are robust.

The subgroup analysis of clinical efficacy and safety did not explore the source of heterogeneity, possibly due to the presence of challenging-to-identify methodological and clinical diversity among these studies. As mentioned earlier, heterogeneity is always present in meta-analyses. Higgins pointed out that as long as the predefined inclusion criteria are met, any degree of heterogeneity is acceptable.^[58]^ Our choice of the random-effects model for analysis aims to make the conclusions more conservative.

Regarding secondary outcome measures, the meta-analysis suggests that the combination of SG and conventional Western medicine can reduce the recurrence rate of the disease (RR = 0.24, 95% CI: [0.14, 0.40], *P* < .00001). There was high homogeneity among the studies (*I*^2^ = 0%). However, this conclusion is based on findings from 5 small-sample original studies, and its reliability awaits further verification. Nonetheless, it provides a potential entry point for future research directions.

The gastrointestinal symptom score is an assessment of the severity of clinical symptoms such as “nausea, vomiting, and epigastric pain” in patients. A lower total score indicates more evident improvement in the patient’s symptoms. The meta-analysis results based on 6 original studies demonstrate that compared to the control group, the treatment group can better alleviate gastrointestinal symptoms in patients (SMD = −1.59, 95% CI: [−2.00, −1.18], *P* < .00001). However, there was significant heterogeneity among the studies (*I*^2^ = 85%). Subgroup analysis was not performed due to the limited number of included original studies. Notably, the original studies did not use the same measurement scale, and the source of heterogeneity may be methodological. Therefore, the analysis results only describe a potential intervention trend, and its true effect requires validation through more rigorous clinical trials.

### 4.2. Reliability of the present findings

This study was conducted following the guidelines outlined in the PRISMA statement. The meta-analysis was registered in the PROSPERO platform, and a meticulously designed literature search strategy was implemented. The quality of the included studies was assessed using RoB2, and appropriate statistical analyses were employed to objectively examine the original research. Additionally, we actively investigated the sources of heterogeneity and performed sensitivity analyses using the leave-one-out method. Publication bias was evaluated using Begg test and Egger test, and quantitative analysis based on the trim-and-fill method was utilized to assess the robustness of the results. By adhering to these standardized meta-analysis procedures, we ensured the reliability and credibility of our findings.

### 4.3. Innovations of the study

This latest meta-analysis aimed to compare the efficacy of SG combined with Western medication with that of Western medication alone in the treatment of FD, with the goal of providing clinicians worldwide with a superior treatment option. Compared with previously published meta-analyses, this study included new RCTs published in the period of 2020 to 2023 and excluded unregulated studies included in previous meta-analyses, such as those without clear diagnostic criteria, with incorrect randomization methods, and with unclear treatment protocols. The present meta-analysis followed the latest guidelines of the PRISMA statement, used a better quality assessment tool (RoB2), and performed a more in-depth exploration of the robustness of the results. Finally, this article was published in English, with the goal of reaching healthcare practitioners worldwide.

### 4.4. Limitations of the study

There are undeniable limitations in the current meta-analysis. Although all included studies were RCTs, pervasive methodological flaws in primary studies necessitate cautious interpretation. Only 13 studies (13/32) specified randomization methods, and critically, no studies reported allocation concealment – a key source of selection bias that may systematically overestimate treatment effects. Similarly, inadequate blinding (implemented in only 2/32 studies) introduces substantial risks of performance and detection bias, further compromising the validity of our efficacy findings.

Geographic generalizability is another major constraint: all studies originated from China. This limits applicability to other populations, as cultural dietary patterns, genetic predispositions, and regional healthcare practices may significantly influence both SG accessibility and treatment outcomes.

For gastrointestinal symptom scores – a critical secondary outcome – limitations are particularly pronounced. Quantitative synthesis included few studies with notable heterogeneity (*I*^2^ = 85%), compounded by non-standardized measurement scales across trials. Given the small sample sizes and methodological inconsistencies, these results represent only a tentative intervention trend and should not be considered definitive. This uncertainty extends to other secondary outcomes, The limited statistical power resulting from small sample sizes significantly amplifies outcome uncertainty—manifested through widened CIs for pooled effect estimates and heightened risk of type II errors. This methodological constraint precludes definitive conclusions regarding these endpoints, necessitating larger, rigorously designed trials to validate our findings.

Finally, while trim-and-fill sensitivity analysis confirmed the robustness of primary efficacy/safety outcomes despite asymmetric funnel plots and positive Egger tests (suggesting possible publication bias), the persistent signal of bias underscores that our pooled estimates might be inflated by unpublished null findings.

Collectively, these limitations demand restrained interpretation of results and highlight an urgent need for multi-regional RCTs featuring standardized outcomes, adequate blinding, allocation concealment, and larger cohorts to verify SG’s clinical profile.

### 4.5. Recommendations for future research

In this meta-analysis, we provided some insights for future research. We recommend that RCTs adhere to the consolidated standards of reporting trials (CONSORT) guidelines to ensure methodological rigor and procedural transparency. Firstly, it is essential that all studies clearly articulate their research objectives, namely, to assess the efficacy of SG as a standalone treatment for FD or in combination with Western medication. Secondly, there should be a clear and explicit description of the randomization methods employed and whether appropriate allocation concealment techniques were utilized. Thirdly, we recommend that RCTs include a placebo control group. Fourth, we suggest that future studies measure a more comprehensive set of outcome measures to enhance the credibility of the research. Finally, sample size estimation based on relevant parameters should be conducted to mitigate potential bias in efficacy assessment.

## 5. Conclusion

The current study, in line with the guidelines specified in the PRISMA statement, reveals that the integration of Shugan Jieyu capsules as a complementary therapy exhibits superior clinical efficacy in the treatment of FD. Notably, the safety profile associated with this combined treatment approach is on par with conventional Western medication therapies. Our study diversifying the therapeutic options for FD and facilitating updates to clinical guidelines. Although funnel plot asymmetry and Egger tests suggest potential publication bias, trim-and-fill sensitivity analyses confirmed the robustness of these efficacy and safety outcomes. However, given the methodological limitations of the original studies – such as the limited number of investigations assessing gastrointestinal symptom scores and the lack of standardized assessment instruments – further rigorous research employing uniform outcome measures is imperative to establish therapeutic efficacy. This underscores the need for high-quality, multicenter RCTs with larger sample sizes to yield robust evidence for clinical decision-making.

## Acknowledgments

We extend our gratitude to all researchers who performed randomized controlled trials (RCTs) included in this study. We also express our appreciation for data analysis software that can be freely accessed online or downloaded for use.

## Author contributions

**Conceptualization:** Shuangli Peng, Yamei Ge.

**Data curation:** Huiyun Pu, Ping Yang.

**Formal analysis:** Shuangli Peng, Hongci Chen.

**Methodology:** Shuangli Peng.

**Project administration:** Hongci Chen.

**Resources:** Hongci Chen.

**Software:** Yamei Ge, Ping Yang, Hongci Chen.

**Supervision:** Huiyun Pu.

**Visualization:** Shuangli Peng, Hongci Chen.

**Writing – review & editing:** Hongci Chen.

## Supplementary Material


