# Supplementary material for: Efficacy and safety of the Shugan Jieyu capsule as a complementary treatment for functional dyspepsia: A systematic review and meta-analysis
Source: Medicine (Baltimore). 2025 Aug 29;104(35):e44058. doi: 10.1097/MD.0000000000044058 (PMC12401241; doi:10.1097/MD.0000000000044058)
Supplement: Supplementary file 1 [file medi-104-e44058-s001.docx]

Supplementary Material S1. Details of the Shugan Jieyu Capsule

| **Formulation** | Shugan Jieyu Capsule |
| --- | --- |
| **Source** | Chengdu Kanghong Pharmaceutical Group or Sichuan Jishitang Pharmaceutical Co., Ltd |
| **SFDA approval number** | Z20174037 or Z20080580 |
| **National Medical Products Administration Standard** | YBZ10382008 |
| **Compositions** | *Hypericum perforatum* L. [Hypericaceae; Hyperici herba], *Eleutherococcus senticosus* (Rupr. & Maxim.) Maxim. [Araliaceae; Acanthopanacis radix et rhizoma seu caulis] |
| **Description** | This product is a hard capsule, the contents of the brown to brown powder, smell fragrant, taste slightly bitter |
| **Extraction procedure** | *Hypericum perforatum* processed with 70% ethanol reflux extraction twice for 1 hour each time, combining the extracted liquids and filtering. The filtrate is vacuum concentrated to a relative density of approximately 1.10 (70°C) to obtain a paste, which is then spray-dried to obtain a dry paste powder. *Eleutherococcus senticosus* is boiled with water three times, each time for 2 hours, combining the decoctions and filtering. The filtrate is concentrated to a relative density of approximately 1.18 (70°C) to obtain a paste, which is also spray-dried to obtain a dry paste powder. Take the dry paste powders of the two ingredients mentioned above, add an appropriate amount of pre-gelatinized starch, 18g of talcum powder, and 2g of magnesium stearate, mix well, fill into capsules, and make 1000 capsules in total. This completes the process. |
| **Dosage and administration** | Oral administration. Take 2 capsules at a time, twice daily, once in the morning and once in the evening. The treatment course is for 6 weeks. |
| **Actions** | Shugan Jieyu, Jianpi Anshen |
| **Indications** | It is used for mild to moderate unipolar depression with a pattern of Ganyu Pixu. Symptoms include low mood, decreased interest, sluggishness, insomnia, vivid dreams, nervousness, irritability, reduced appetite, chest tightness, fatigue, excessive sweating, pain, with a white or greasy tongue coating, and a pulse that may be wiry or thin |
| **Storage** | Seal |
| **Quality control reported? (Y/N)** | Y-prepared according to the National Medical Products Administration Standard YBZ10382008 |
| **Chemical analysis reported?**  **(Y/N)** | Y-HPLC, Detail information can be got from Pharmacopoeia of the People's Republic of China(2000) (Part I) |

Supplementary Material S2. Key active ingredients in Shugan Jieyu Capsule

The active ingredients of the Shugan Jieyu capsules were obtained using the Traditional Chinese Medicine Systematic Pharmacology Database and Analytical Platform (TCMSP) at https://tcmsp-e.com/ (screening conditions: bioavailability (OB) ≥ 30% and drug-likeness (DL) ≥ 0.18), or they were obtained from The Encyclopedia of Traditional Chinese Medicine (http://www.tcmip.cn/ETCM). As shown in the table below, the active ingredients of *Hypericum perforatum* were obtained from the TCMSP database, while the active ingredients of *Acanthopanax senticosus* were obtained from the ETCM database.

| Compositions of Runzao Zhiyang Capsule | Chemicals | Reource |
| --- | --- | --- |
| *Hypericum perforatum* L. [Hypericaceae; Hyperici herba] | sitosterol | TCMSP |
|  | kaempferol | TCMSP |
|  | (+)-catechin | TCMSP |
|  | luteolin | TCMSP |
|  | ent-Epicatechin | TCMSP |
|  | quercetin | TCMSP |
| *Eleutherococcus senticosus* (Rupr. & Maxim.) Maxim. [Araliaceae; Acanthopanacis radix et rhizoma seu caulis] | 3-O-trans ferulylquinic acid | ETCM |
|  | Sitosterol,Î’-Sitosterol | ETCM |
|  | Caffeic Acid | ETCM |
|  | Isofraxidin,Phytodolor | ETCM |
|  | Ethyl Oleate | ETCM |
|  | Cinnamic Acid,Hydroxycinnamic Acid,P-Coumaric Acid,P-Hydroxy Cinnamic Acid | ETCM |
|  | Vanillin | ETCM |
|  | 3,4-Dihydroxybenzaldehyde,Hydroxybenzoic Acid,M-Hydroxybenzoic Acid,P-Hydroxybenzoic Acid,Salicylic Acid | ETCM |
|  | Octadecanoic?Acid,Stearic Acid | ETCM |
|  | Oleanolic Acid | ETCM |
|  | Alexandrin,Daucosterol,Eleutheroside A | ETCM |
|  | Sucrose | ETCM |
|  | Alexandrin,Daucosterol,Caproic Acid,Eleutheroside A,Sitogluside,Strumaroside,Î’-Sitosterol-Î’-D-Glucoside | ETCM |
|  | 3,4-Dihydroxybenzoic Acid,Protocatechuic Acid | ETCM |
|  | Betulinic Acid | ETCM |
|  | Sesamin | ETCM |
|  | Syringin | ETCM |
|  | Ursolic Acid | ETCM |
|  | Syringaresinol | ETCM |
|  | Syringaresinol-Di-O-Î’-D-Glucoside | ETCM |
|  | Cedar Acid,Syringic Acid | ETCM |
|  | 9,11-Octadecadienoic Acid | ETCM |
|  | 7Z,10Z,13Z-Hexadecatrienoic Acid,Hexadecatrienoic Acid | ETCM |
|  | Sinapaldehyde Glucoside | ETCM |
|  | Coniferin | ETCM |
|  | Coniferaldehyde,Coniferyl Aldehyde,Trans-Coniferyl Aldehye | ETCM |
|  | Coniferaldehyde Glucoside | ETCM |
|  | Eleutheroside C | ETCM |
|  | Chlorogenic Acid | ETCM |
|  | Hyperin,Hyperoside,Hyperoside,Quercetin-3-O-Galactoside | ETCM |
|  | Methyl Oleate | ETCM |
|  | Ciwujianoside B | ETCM |
|  | Ciwujianoside D2 | ETCM |
|  | Eleutheroside L | ETCM |
|  | Ciwujianoside E | ETCM |
|  | Alpha-Pinene,Amygdalin | ETCM |
|  | 3-O-Î‘-L-Arabinopyranosyloleanolic Acid | ETCM |
|  | Ciwujianoside C1 | ETCM |
|  | Ciwujianoside D1 | ETCM |
|  | Ciwujiatone | ETCM |
|  | (1R)-1,11-Î‘-Dihydroxy-3,4-Seco-Lupa-4(23), 20(29)-Diene-3,28-Dioic Acid 3,11-Lactone 28-O-Î‘-L-Rhamnopyranosyl-(1â†’4)-Î’-D-Glucopyranosyl(1â†’6)-Î’-D-Glucopyranoside | ETCM |
|  | 2,6-Dimethoxybenzoquinone | ETCM |
|  | Eleutheroside K | ETCM |
|  | 1-Ethyl-Î‘-D-Galactoside | ETCM |
|  | Hederagenin-3-O-Î’-Glucuronopyranoside | ETCM |
|  | Isofraxidin Glucoside | ETCM |
|  | Isolinolic Acid | ETCM |
|  | Liriodendrin | ETCM |
|  | (+)-Medioresinol Di-O-Î’-D-Glucopyranoside | ETCM |
|  | 10,13-Octadecadienoic Acid Ethyl Ester | ETCM |
|  | (+)-Pinoresinol-Di-O-Î’-D-Glucoside | ETCM |
|  | 3Î’-{O-Î‘-L-Rhamnopyranosyl-(1â†’4)-O-Î‘-L-Rhamnopyranosyl-(1â†’4)-[O-Î‘-L-Rham-Nopyranosyl-(1â†’2)-O-Î’-D-Glucopyranosyl(1â†’4)-O-Î’-D-Glucuronopyranosyl]}-16Î‘-Hydroxy-13Î’, 28-Epoxyoleanane | ETCM |
|  | Akeboside Sth | ETCM |
|  | Chiisanogenin | ETCM |
|  | Chiisanoside | ETCM |
|  | Eleutheroside I | ETCM |
|  | Neociwujiaphenol | ETCM |

**Supplementary Material S3. Search strategies for databases**

**Pubmed**

Search:(((((((functional dyspepsia) OR (postprandial distress syndrome)) OR (epigastric pain syndrome)) OR (FD)) OR (PDS)) OR (EPS)) AND ((Shuganjieyu) OR (Shugan Jieyu))) AND ((randomized controlled trial[pt] OR controlled clinical trial[pt] OR randomized[tiab] OR placebo[tiab] OR clinical trials as topic[mesh:noexp] OR randomly[tiab] OR trial[ti]) NOT (animals[mh] NOT (humans[mh] AND animals[mh]))) AND (("2008/10/20"[Date - Publication] : "2024/01/01"[Date - Publication]))

#1 functional dyspepsia

#2 postprandial distress syndrome

#3 epigastric pain syndrome

#4 FD

#5 PDS

#6 EPS

#7 #1 OR #2 OR #3 OR #4 OR #5 OR #6

#8 Shuganjieyu

#9 Shugan Jieyu

#10 #8 OR #9

#11 (randomized controlled trial[pt] OR controlled clinical trial[pt] OR randomized[tiab] OR placebo[tiab] OR clinical trials as topic[mesh:noexp] OR randomly[tiab] OR trial[ti]) NOT (animals[mh] NOT (humans[mh] AND animals[mh]))

#12 ("2008/10/20"[Date - Publication] : "2024/01/01"[Date - Publication])

#13 #7AND #10 AND #11 AND #12

**Embase**

#1 'functional dyspepsia'/exp OR 'functional dyspepsia' OR (functional AND ('dyspepsia'/exp OR

dyspepsia))

#2 'postprandial distress syndrome' OR 'epigastric pain syndrome' OR 'fd' OR 'pds' OR 'eps'

#3 'shuganjieyu' OR 'shugan jieyu'

#4 'crossover procedure':de OR 'double-blind procedure':de OR 'randomized controlled trial':de OR 'single-blind procedure':de OR (random* OR factorial* OR crossover* OR cross NEXT/1 over* OR placebo* OR doubl* NEAR/1 blind* OR singl* NEAR/1 blind* OR assign* OR allocat* OR volunteer*):de,ab,ti

#5 ' [2008-10-20] : [2024-01-01] '

#6 #1 AND #2 AND #3AND #4 AND #5

**Cochrane Library**

#1 (functional dyspepsia):ti,ab,kw (Word variations have been searched)

#2 (postprandial distress syndrome):ti,ab,kw OR (epigastric pain syndrome):ti,ab,kw (Word variations have been searched)

#3 (FD):ti,ab,kw OR (PDS):ti,ab,kw OR (EPS):ti,ab,kw (Word variations have been searched)

#4 (Shuganjieyu):ti,ab,kw OR (Shugan Jieyu):ti,ab,kw (Word variations have been searched)

#5 Date: 20 October 2008 to 01 January 2024

#6 #1 AND #2 AND #3 AND #4 AND #5

**China National Knowledge Infrastructure database search strategy**

(主题=舒肝解郁) AND (主题=消化不良) AND 时间范围：2008-10-20~2024-01-01

**Wan Fang database search strategy**

(主题:(舒肝解郁) and 主题:(消化不良)) and Date:2008-2024*

**VIP database search strategy**

任意字段=舒肝解郁AND任意字段=消化不良AND年份：2008-2024

**China Biomedical Literature Service System**

("舒肝解郁"[常用字段] AND ("消化不良"[常用字段] OR "胃弱"[常用字段] OR "消化障碍"[常用字段] OR "消化不良"[主题词])) AND (临床试验[文献类型] OR 随机对照试验[文献类型] OR 多中心研究[文献类型]) AND 人类[特征词] AND 2008-2024
